# Spinal cord lesions shrink peripersonal space around the feet, passive mobilization of paraplegic limbs restores it

**DOI:** 10.1038/srep24126

**Published:** 2016-04-06

**Authors:** Michele Scandola, Salvatore Maria Aglioti, Claudio Bonente, Renato Avesani, Valentina Moro

**Affiliations:** 1 NPSY-Lab.VR, Department of Human Sciences, University of Verona, Verona I-37129, Italy; 2 IRCCS, Fondazione Santa Lucia, Rome I-00179, Italy; 3 Department of Psychology, University of Rome “Sapienza”, Rome I-00185, Italy; 4 Department of Rehabilitation, Sacro Cuore - Don Calabria Hospital, Negrar I-37024, Verona, Italy

## Abstract

Peripersonal space (PPS) is the space surrounding us within which we interact with objects. PPS may be modulated by actions (e.g. when using tools) or sense of ownership (e.g. over a rubber hand). Indeed, intense and/or prolonged use of a tool may induce a sense of ownership over it. Conversely, inducing ownership over a rubber hand may activate brain regions involved in motor control. However, the extent to which PPS is modulated by action-dependent or ownership-dependent mechanisms remains unclear. Here, we explored the PPS around the feet and the sense of ownership over lower limbs in people with Paraplegia following Complete spinal cord Lesions (PCL) and in healthy subjects. PCL people can move their upper body but have lost all sensory-motor functions in their lower body (e.g. lower limbs). We tested whether PPS alterations reflect the topographical representations of various body parts. We found that the PPS around the feet was impaired in PCL who however had a normal representation of the PPS around the hands. Significantly, passive mobilization of paraplegic limbs restored the PPS around the feet suggesting that activating action representations in PCL brings about short-term changes of PPS that may thus be more plastic than previously believed.

The space immediately around the body (i.e. the region of space within which objects can be grasped and manipulated) is known as the Peripersonal space (PPS). Electrophysiological experiments on monkeys and neuropsychological and neuroimaging studies on humans (for a review see^1^) showed that there are complex neuronal networks in premotor, parietal and subcortical brain regions underlying PPS^2^,^3^,^4^,^5^,^6^,^7^. A fast, automatic integration of various different sensory and motor signals within the PPS is guaranteed by multisensory bimodal neurons (e.g. visuo-tactile, audio-tactile and visuo-motor) or even trimodal neurons (e.g. audio-visuo-motor)^8^. Moreover, PPS is organized according to coordinated head-9, hand-10,11, trunk-12,13,14,15 and feet-centred^16^,^17^ systems that exhibit different levels of interaction^16^.

It has been acknowledged that PPS may be modulated (extended or reduced) by a number of factors related to plasticity. For example, the extensive use of a computer mouse enlarges the PPS around the hand and this enlargement may include the screen monitor, at least during the time when the hand remains in contact with the mouse^18^. Furthermore, in the case of blind people, regular use of a cane to navigate extends their PPS to the full length of the cane^19^. Similar plastic changes have been recorded in healthy people after brief training sessions with a cane^19^ or two sticks, in particular when active movements with tools are requested^20^. In the same way, if a limb is forced to immobilization for 10 hours, a reduction in the extent of the PPS occurs^21^. Although these results indicate that PPS boundaries are influenced by actions performed within it, changes can also be noted if a rubber hand is placed in a position which is coherent with the person’s body^8^,^22^. Furthermore, the PPS around the fake hand extends in direct correlation to the intensity of the sense of ownership towards the rubber hand^23^,^24^. Thus, the sense of ownership or the embodiment of a tool (e.g. a prosthesis in amputees^25^) might be sufficient to enhance a representation of PPS.

However, although *action-dependent* and *embodiment-dependent* mechanisms may explain the plasticity of PPS, the specific impact of these mechanisms remains unclear.

For example, in upper-arm amputees, PPS boundaries typically shift towards the stump. However, when these patients wear a functional prosthesis the shift extends in order to include the prosthetic hand^26^. This effect may be explained by both *action-* and *embodiment-dependent* mechanisms. In fact, the prosthesis reproduces the exterior appearance of a real arm and hand and, at the same time, allows actions to be performed since the prosthesis is myoelectrically or kinematically controlled by residual muscles.

In order to tease apart the specific role of the above mechanisms in remapping PPS, we tested people with spinal cord injury (SCI), a condition that leads to a more or less complete body-brain disconnection and a loss of sensory and motor functions below the lesion leaving the appearance of the body, however, unchanged. This condition might potentially involve a dissociation between PPS and the sense of ownership of the paralyzed limbs.

Previous research has shown that SCI induces brain plasticity^27^,^28^,^29^,^30^,^31^ with changes in bodily representations^32^,^33^, in motor imagery^34^, in mental rotation of body parts^35^ and in the visual discrimination of body actions and forms^36^. Interestingly, these changes seem to follow somato-topic rules^32^,^34^,^35^,^36^.

In order to assess the impact of lower limb de-afferentation and de-efferentation on PPS boundaries in the absence of changes in body form, we used a modified version of the Crossmodal Congruency Task (CCT)^37^,^38^. In its original version the task required participants to discriminate the position of tactile stimuli (administered in a “high” or “low” position in a tactile-stimuli-delivering device) administered to their hands, at the same time attempting to ignore a series of visual distractors that were activated close by or relatively distant (i.e., ipsilaterally or contralaterally) from the hands which had been placed in a congruent or incongruent elevation, with respect to the tactile stimulus. Reaction Times (RT) were only slowed down by the distractors when the stimuli were Ipsilateral and Incongruent (as compared to Ipsilateral and Congruent) and when they were either close to or within the PPS around the hands. The difference between the RTs obtained in Incongruent and Congruent trials offers a measure of the Crossmodal Congruency Effect (CCE). In our task a Contralateral CCE (the difference between Incongruent and Congruent Contralateral trials) and an Ipsilateral CCE (the difference between Incongruent and Congruent Ipsilateral trials) are computed. While Contralateral CCEs occur in purely spatial-perceptive tasks, Ipsilateral CCEs are also influenced by PPS interference. Thus, a PPS representation is postulated if the Ipsilateral CCEs are greater than the Contralateral CCEs^20^,^37^.

In this study, we used a CCT that enabled us to investigate the integration between the PPS around the feet and tactile sensations in the hands^16^. Participants held two tactile target stimulators (one in each hand) while distractor lights were inserted in a wooden frame placed in a position which was coherent with their lower limbs. In this way, we could compare the CCE induced by distractor lights positioned near their real feet (i.e., the feet within the wooden frame) and CCE induced by distractor lights placed in the same spatial location but relatively far from their feet (i.e., the feet were outside the wooden frame). Furthermore, to test if the presence of fake legs could evoke a PPS representation (analogous to the rubber hand experiment), we also introduced a condition with a pair of fake feet. We applied this procedure to three experimental designs. In the first one, we tested the PPS around the feet in a group of people affected by Paraplegia with Complete Lesion (PCL). This was compared to gender - and age -matched healthy controls (Group C). Furthermore, the sense of embodiment of paralyzed limbs was measured by means of an ad-hoc questionnaire (modified from^39^). A second experiment allowed us to check whether any changes in PPS specifically involved the region of space around the paralyzed limbs or also involved the PPS around non-disconnected body parts (e.g. around the hands). Finally, in Experiment 3 we tested the role of passive motion with the aim of restoring the PPS around the feet in two PCL groups.

## Results

Reproducibility problems have very recently arisen in research studies in the field of psychology^40^. This also highlights the weakness of p-values in terms of guaranteeing the replicability of results^41^. Furthermore, frequentist statistics does not allow the Null hypothesis to be verified. For these reasons, we used a Bayesian Model Comparison by means of a transdimensional Monte Carlo Markov chain known as the Space Product Method (SPM)^42^. By means of the SPM, a series of models representing various hypotheses were tested. Using a categorical index encompassing the models, we obtained the number of times which the models were visited to account for the data observed. These proportions are indicated by 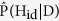, with id being the hypothesis identifier. These values range from 0 to 1 (maximum probability), giving an intuitive measure of which hypothesis is more trustworthy. The priors are described in the Supplementary Materials (SM, Figures S1 and S2).

To remain within the mainstream framework of frequentist statistics, standard ANOVAs and ordered logistic regressions were also run (see SM, S3). It is worth noting that although different, the SPM and the frequentist approaches provided identical results where comparable (i.e. not in the acceptance of the null hypothesis).

### Experiment 1: Paraplegia modifies PPS around the feet

**Figure 1:**
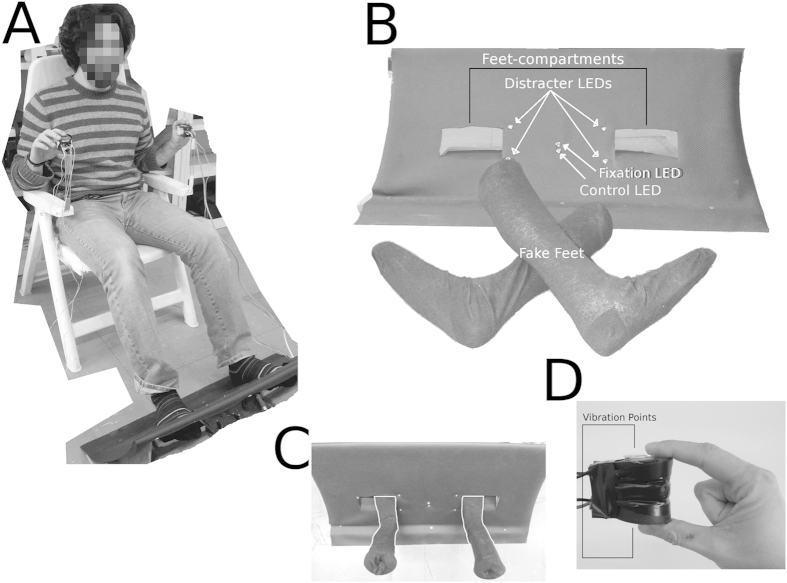
(**A**) Experimental set up. Typical position of a participant for the Real condition in Experiment 1 and Experiment 3. Note the participant’s feet within the feet-compartments of the wooden frame. (**B**) Wooden frame with fake feet and LEDs position. (**C**) Wooden frame with fake feet inserted into the feet compartments. (**D**) Tactile stimulators where vibration is delivered against the index (high position) or the thumb finger (low position).

In order to assess the PPS around the feet in participants who cannot feel any tactile sensations in their feet (or perform any movement with those body parts), we applied a visuo-tactile CCT similar to Schicke’s experiment^16^. Visual distractors were placed (see Fig. 1B) on a wooden frame near two empty holes for feet (feet-compartments) designed so that the feet of the participant could be comfortably inserted. Tactile target stimuli were applied to the index finger and the thumb of both hands (Fig. 1D). All participants (18 healthy controls (C) and 18 PCL participants, see Table 1 for demographic and clinical details) sat on a chair in front of the wooden frame. Three conditions were used in which the participant saw: i) their own feet wearing black socks (Real condition); ii) two fake feet wearing the same socks (Fake condition) and iii) nothing (Void condition). The order of the conditions was counterbalanced across participants. Each condition was divided into three blocks (162 trials, 9 trials for each combination of visuo-tactile stimulation - total n.144, n.9 control and n.9 fake stimulations). We computed the Ipsilateral and Contralateral CCEs from the RTs of correct trials.

At the beginning and end of each block, participants filled in a questionnaire assessing their Sense of Embodiment, Loss of their own feet, Perceived Movement of lower limbs and Compliance (see Methods for further details and S4.2 for the list of questions). A 5′ pause was allowed after each block.

### CCE Analysis

We analysed 8 hypotheses for each group by means of SPM (represented by hierarchical linear Bayesian models, for more details see SM S1) going from an absence of the PPS effect to the presence of the PPS effect in all conditions. The absence of the PPS effect was when the difference between Ipsilateral and Contralateral CCEs was 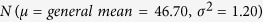 distributed. 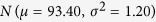 indicated the presence of a PPS effect.

**Figure 2:**
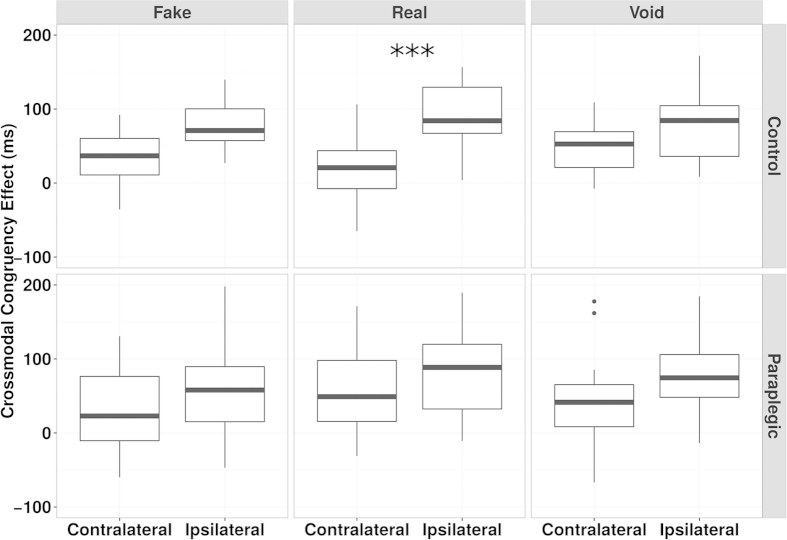
Experiment 1 - CCE around the feet, Boxplot of CCE data. The graph is organised as a matrix: the Conditions (Fake, Real, Void) are in the columns and the groups (Control group and Paraplegic group) are in the horizontal rows (top and bottom). Contralateral and Ipsilateral data are side by side in order to allow direct comparisons. The darker line in the middle of the box is the median, the box represents the first and the third quartile and the bottom and top whiskers represents respectively the first quarter minus 1.5 * Interquartile Range and the third quarter plus 1.5 * Interquartile Range. The means and standard deviations for the CCE of the Control group are: Fake Contralateral = 35.43 ± 32.78; Fake Ipsilateral = 77.52 ± 31.65; Real Contralateral = 20.56 ± 41.53; Real Ipsilateral = 92.86 ± 43.49; Void Contralateral = 47.62 ± 33.83; Void Ipsilateral = 77.86 ± 41.66. The means and standard deviations for the CCE of the Paraplegic group are: Fake Contralateral = 29.38 ± 53.21; Fake Ipsilateral = 81.93 ± 88.91; Real Contralateral = 52.43 ± 57.79; Real Ipsilateral = 88.96 ± 63.71; Void Contralateral = 46.73 ± 57.67; Void Ipsilateral = 90.88 ± 62.89. The box with “***” represents the condition where Contralateral and Ipsilateral CCEs were different according to the SPM and frequentist (see SM, S4.1) statistics.

In Group C, results show the presence of PPS exclusively in the Real condition (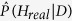 = 0.73), while in PCL participants we found no differences between the conditions (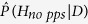 = 0.79). Figure 2 shows a graphical representation of the data.

### Questionnaire Responses

**Figure 3:**
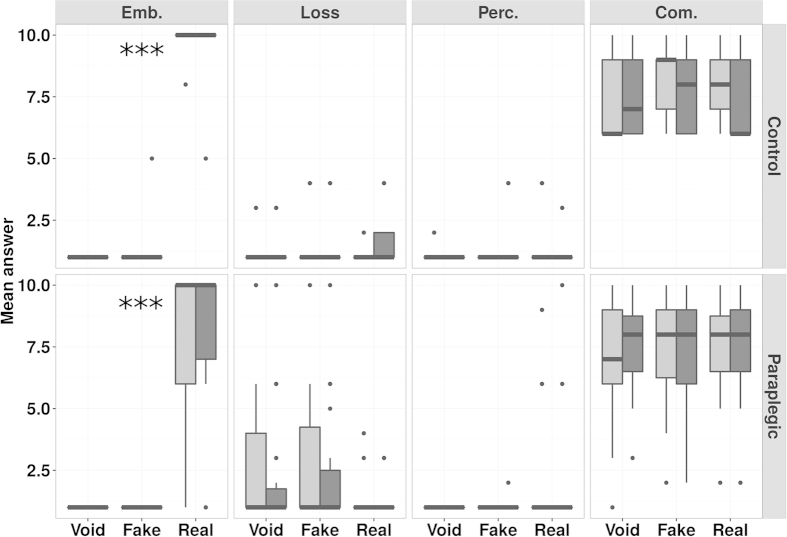
Experiment 1 - CCE around the feet, Boxplot of questionnaire data. The graph is organised in a matrix: the Components (Emb. = Embodiment of own feet, Loss = Loss of own feet, Perc. = Perceived Movements and Com. = Compliance) are in the columns and the groups (Control group and Paraplegic group) are in the horizontal rows (top and bottom). The timing (Before – lighter grey and After – darker grey) and the experimental conditions (Fake, Real, Void) are represented side by side for ease of comparison. The values are as in Fig. 2. The boxes with “***” represent the conditions where there were different subjective “Embodiment of own feet” sensations among conditions.

For each group, 22 different hypotheses were analysed for each component of the questionnaire with regard to the sensations which had been tested by means of SPM. These hypotheses were computed by means of hierarchical logit Bayesian models (for further details see SM S2). Logit model coefficients are the logarithm of odds ratios, therefore we postulated that high values follow the distribution 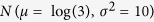, while low values follow the distribution 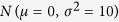. The results show elevated embodiment sensations in the C and PCL groups, both before and after the Real condition (
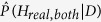
 = 0.98, 
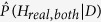
 = 0.87, respectively, Fig. 3). Compliance was high for both groups in all conditions (C group: 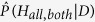 = 0.81, PCL group: 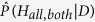 = 0.52, for all the remaining hypotheses 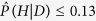). Perceived Movement was low in all conditions in both groups (PCL group: 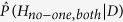 = 0.97, C group: 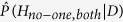 = 0.95). The same result was observed for sensations of Loss of own feet (PCL group: 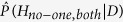 = 0.92, C group: 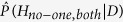 = 0.96) (see Fig. 3 and Table 2). In all cases the ownership scores were not different after and before the task. This suggests the PPS does not change any of the subjective sensations assessed by our ad-hoc questionnaire.

To sum up, the results of this experiment indicate that although PCL and C participants did not show any differences in the explicit sense of ownership of their own legs , the former group lose implicit representations of PPS around the feet.

### Experiment 2: Paraplegia does not affect the PPS centred around the hands

14 people with PCL participated in this experiment concerning the PPS around the hands. The procedure was similar to Experiment 1 but the compartmentalised wooden framework was placed on a table in front of the participants who were seated in a comfortable position. There were two separate conditions: participants saw their hands within the compartments (the “Within compartments” condition) or they kept their hands near their own body while the compartments were out of their reach (the empty compartments, “Outside compartments” condition). The number of trials for each condition was the same as in Experiment 1.

Using hierarchical Bayesian linear models for each group, we analysed 4 hypotheses by means of the SPM, starting from a general absence of PPS and ending with the presence of PPS in all conditions. The absence of a PPS effect is 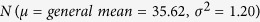 distributed, while the presence of a PPS effect is 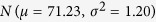 distributed (see SM S1).

**Figure 4:**
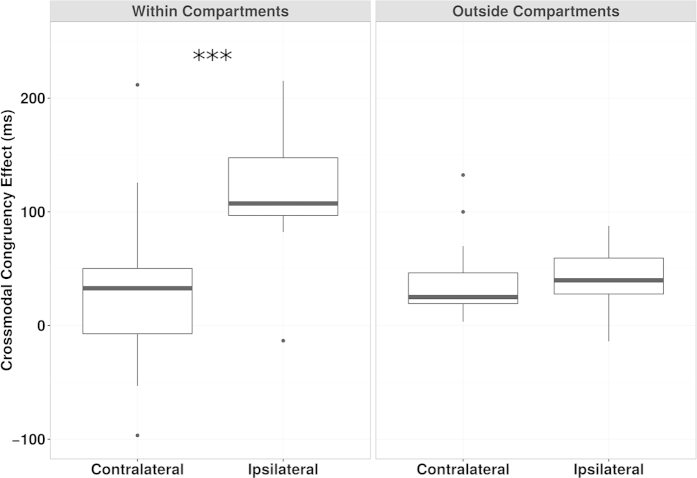
Experiment 2 - CCE around the hands, Box plot of CCE data. The graph is organized as a matrix: the Conditions (Within Compartments, Outside Compartments) are in the columns, Contralateral and Ipsilateral data are side by side in order to facilitate interpretation. Means and standard deviations for the CCE: Within Compartments Contralateral = 36.94 ± 77.89; Within Compartments Ipsilateral = 122.88 ± 58.99; Outside Compartments Contralateral = 57.21 ± 132.16; Outside Compartments Ipsilateral = 40.51 ± 88.96. The box with “***” represents the condition where Contralateral and Ipsilateral CCEs were different according to the SPM and frequentist (see SM, S4.1) statistics.

The results confirm that the PPS around the hands is preserved in the PCL participants (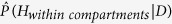 = 1) (see Fig. 4).

### Experiment 3: Passive motion of the lower limbs restores the PPS around the feet in paraplegics

Following the same general procedure, two groups randomly selected from the PCL participants in Experiment 1 were compared. In the first group (the Motion group, n.7), the participant’s legs were passively moved for 5 minutes (3 blocks, for a total of 15′) by an experimenter and a physiotherapist before each session of visuo-tactile stimulation. Short-term modulations have been previously proved to be effective in modulating both embodiment and PPS^19^,^32^,^33^. This consisted of slow alternate movements involving flexo-extension of the knees. These movements were enough slow to avoid spastic reactions. The range of joint motion was complete because participants did not have structural retractions. The other participants (the No-motion group, n.7) just had a conversation with the experimenters for 5′ (for a total of 15′). By means of the SPM, we analysed their performance in Experiment 1 in order to check whether (in a baseline condition) the two groups did not differ each other and lacked PPS around the feet.

The four hypotheses we analysed were: i) the absence of PPS in both groups; ii) the presence of PPS in the Motion group; iii) the presence of PPS in the No-motion group and iv) the presence of PPS in both groups. The presence or absence of PPS were expressed as reported for Experiment 1. Results showed an absence of PPS in both groups (Motion group, all conditions: 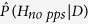 = 0.997; No-motion group, all conditions: 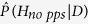 = 1).

**Figure 5:**
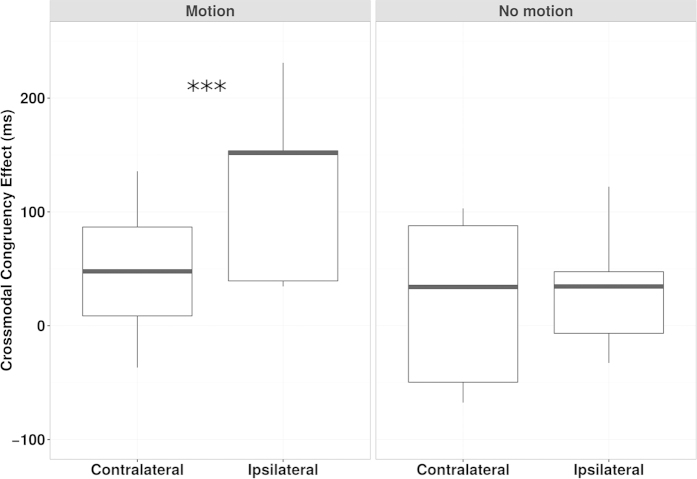
Experiment 3 - CCE around the feet after passive motion, Box plot of CCE data. The graph is organised in columns with the Motion group on the left and the No-motion group on the right. Contralateral and Ipsilateral data are side by side in order to facilitate interpretation. Means and standard deviations for the CCE: Motion Contralateral = 48.19 ± 60.30; Motion Ipsilateral = 114.78 ± 77.19; No-motion Contralateral = 20.87 ± 75.46; No-motion Ipsilateral = 0.43 ± 96.76. The box with “***” represents the condition where Contralateral and Ipsilateral CCEs were different according to the SPM and frequentist (see SM, S4.1) statistics.

In Experiment 3 we analysed the same hypotheses by means of the SPM (presence of PPS = 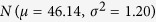, absence = 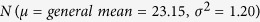). We found that the hypothesis that in the No-motion group both the average CCE values for the Contralateral and the Ipsilateral trials are equal to zero is true (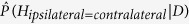 = 1). In contrast, the most probable hypothesis for the Motion group regarded a difference in CCE between the Contralateral and Ipsilateral trials (Ipsilateral trials greater than Contralateral trials, 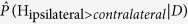 = 1) (Fig. 5).

These results suggest that passive motion of lower limbs in Paraplegics brings about a restoration of the representation of PPS around the feet.

## Discussion

Using a cross-modal integration paradigm, we explored whether the somato-sensory and motor disconnection that characterises spinal cord lesions alters the representation of PPS and whether any alterations specifically affect the space around the disconnected body parts. Four main, potentially important findings came to light: (a) PPS representation around the body parts affected by de-afferentation and de-efferentation (i.e. the feet) is severely affected in people who have suffered SCI; (b) no changes in the PPS around the hands were observed, suggesting a topographic specificity of the effect found for lower limbs; (c) passive mobilisation restores the representation of PPS around the feet, probably due to the recruitment of short-term plasticity mechanisms and (d) the sense of ownership of the legs was spared in PCL participants suggesting that the loss of PPS around the feet may not be associated with general changes in body representations. Taken together, these results confirm that PPS representations are highly alterable and that the sensory-motor connections between the body and the brain play a fundamental role in modulating the perception of PPS. Long-term mechanisms of plasticity after traumatic SCI involve the sensory-motor systems as well as the high cognitive functions related to body and space representations^32^,^36^. Nevertheless, these representations maintain a certain degree of plasticity as demonstrated by the effects of the short-term plasticity induced by passive motion.

Results from Experiments 1 and 2 show that paraplegic people suffer a loss of representation of PPS as a result of long term de-efferentation/de-afferentation. This alteration in PPS follows somato-topic rules. In fact, in paraplegics with complete lesions, PPS disappears specifically around their lower limbs but is preserved around their hands. This indicates that afferences and efferences play an important role in modulating space representation and sheds new light on the debate regarding the mechanisms underlying PPS representation. However the completeness of the lesion in our patients was exclusively assessed by means of a clinical measure (ASIA scale). There is the possibility that, despite the absence of sensorial feedbacks and motor control, an anatomical bridge is still present and that this transmits sensations (that do not reach the level of consciousness) to the brain, as well as motor commands insufficient to start or control any movement.

Studies in non-human and human primates have shown that in order to assess PPS the integration of multisensory stimuli occurring on or close to the body is crucial. This integration is likely subserved by bimodal cells in the posterior parietal cortex, the premotor cortex and the putamen^3^,^23^,^43^. Bimodal and trimodal cells are characterised by the fact that they respond equivalently to multimodal stimuli presented within their receptive field^7^,^44^. Thus, multisensory stimuli occurring around the body will be processed in a integrated way, inducing detailed processing (see detailed evaluation theory^45^). Seminal neurophysiological studies on monkeys have shown separate bimodal neuronal responses for head-, hand- and trunk-centred PPS representations^44^,^46^,^47^,^48^,^49^. To date, only few studies have directly tested the existence of a representation of PPS around the feet. From an evolutionary point of view, the existence of a PPS for the feet is a plausible hypothesis as in monkeys and in our common ancestors the distinction between the feet and the hands and their functional use are not as strong as in humans^48^,^49^.

Schicke and colleagues^16^ first provided evidence to support this hypothesis in humans. They studied the PPS representation surrounding the hands and feet and the interaction between these representations in healthy subjects. No differences between the PPSs around the hands and the feet were found. In addition, the authors reported the existence of an interaction between these two PPSs. Alternating crossed and uncrossed limb postures, Van Elk and colleagues^17^ found a PPS representation around the feet. Nevertheless, only the PPS around the hands (but not the feet) was dynamically modulated by the position of the limbs. In line with Schicke^16^ and Van Elk’s^17^ findings, our results in Experiment 1 support the existence of a PPS representation around the feet in able-bodied humans. However, we did not find the PPS effects in the fake feet condition in the healthy group reported by Van Elk and colleagues^17^. This may be due to a simple but important difference between the two experimental paradigms. In our experiment, the disconnection between the participant’s body and the fake feet was visible and no attempt was made to evoke a sense of ownership with respect to the fake feet. In contrast, in Van Elk and colleagues’^17^ study, a blanket was used to cover the separation between the fake feet and the participant’s body, a condition that may have evoked an embodiment of the fake feet, as the studies concerning the relation between body-continuity and embodiment suggest^50^,^51^.

One potentially important result of our study regards the somato-topographical organization of the changes in PPS representation. In fact, although people affected by PCL lose the PPS around their feet, they maintain it around their hands. Neuroplastic modifications following SCI do not only involve the somato-sensory and motor systems but also extend for example to the visual modality^36^ or higher-order cognitive functions such as those underpinning the sense of ownership^32^. We demonstrated, for example, that people affected by paraplegia show specific difficulties in discriminating body form and body actions when the differences regard the lower limbs but not the upper limbs^36^. Conversely, people affected by PCL who regularly perform sport become more expert than healthy people and PCL patients who do not practise sport when discriminating upper body parts^36^. Moreover, somato-topographic changes concerning the sense of ownership and motor imagery have been demonstrated in people with SCI^32^,^34^. Thus, somatic afferences and motor efferences play a crucial role in building, maintaining and adapting the representations of one’s own body and in all the high cognitive functions which are connected in some way to body perception and action.

Another novel result from our study is that the application of a short term passive mobilization of the disconnected lower limbs brought about a recovery of PPS representation (Experiment 3), indicating that even passive action may modulate PPS. That short term manipulation may be sufficient to modify PPS boundaries has been demonstrated in previous studies^20^,^52^,^53^. Forced immobilization of the arm for 10 hours in healthy people^21^ and the amputation of an arm^26^ both reduce PPS representation. Interestingly, while the over-use of an arm does not seem to modulate PPS^21^ the use of a prosthesis in an amputee sample induces a recovery of the PPS representation around the prosthesis^26^. However, it is not clear if the effect found in amputees is due to the functional use of the prosthesis or to its embodiment. Our results showing that passive movement (but not embodiment) restores PPS representation suggest that the functional use of a prosthesis may activate motor areas^54^, enlarging or restoring PPS representation. This supports the hypothesis regarding “action-dependent” PPS modulation.

The restorative effect of passive motion on the PPS around the feet may be the effect of multiple factors, such as visual feedback, interoception, indirect supralesional sensorial feedback or even residual subclinical somatic sensations transmitted from the legs to the brain.

Our results on the effects of passive mobilization seem to be in contrast with previous research indicating that active motion and utilization are effective in extending PPS^20^ but passive movement is not. In fact, passive holding of tools^55^,^56^, even for prolonged periods^57^, did not modulate the PPS of healthy subjects. However, other studies^22^,^58^ found that placing a fake rubber hand in a position that is congruent with the participants’ body may modulate the representation of PPS. Similar results were confirmed with fake legs^17^. This suggests that in order to extend the PPS, training with tools is required to stimulate the neuroplasticity of action representations. In contrast, dummy body parts may automatically extend PPS representation if the dummy body part are placed in a position which is coherent with the body^22^,^26^. In individuals affected by long-term body-brain disconnection, such as the PCL participants in our study, a brief training session is required to recall the action representation. However, only further studies will reveal the roles of visual appearance, passive and active movements, afferences from mobilized body parts and indirect afferences from other non-mobilised body parts as well as of top-down factors.

In Experiment 1 we observed that the sense of ownership (resulting from a self-reporting questionnaire) is dissociated from PPS (assessed by the presence or absence of CCE). This contrasts studies where a relationship between the sense of ownership and the CCE was reported^24^,^59^,^60^. For example, in these studies, the CCE, the subjective sense of embodiment and the drift (the perceived shift of the body part towards the dummy body part) were all larger when the Full Body Illusion^59^ and Rubber Hand Illusion^24^,^60^ were stronger. In our Paraplegic group however (Experiment 1), higher rates of ownership were reported for the Real Feet condition than the other conditions, but no CCE was found. Conversely, in our group of healthy subjects the CCE and the referred sense of ownership were strictly related.

A dissociation between the mechanisms underlying PPS and body representations was suggested by Bassolino and colleagues in their study^21^. Immobilizing an arm induced a reduction in the representation of PPS but it did not affect the metric representation of the limb (measured as the perceived length of the limb). On the other hand, these authors found that overuse of the arm modulated the metric representation, leaving the PPS around the limb unchanged (for other data concerning dissociation, see^32^,^61^). As PPS and body representation are probably the result of an integration between body sensory perception, action and top-down cognitive mechanisms, we suggest that these features play a different role and have different influences in daily life situations. The balance between them can also change in specific neurological conditions, such as de-afferentation and de-efferentation after SCI.

## Conclusion

PPS appears to be highly dynamic and somato-topically organized. In paraplegics affected by complete lesions, PPS is massively reduced around the ‘disconnected’ lower limbs, even in the presence of a subjective sense of ownership. Importantly, PPS is spared for the hands. Moreover, the passive motion of lower limbs for 15′ can restore a short term PPS representation. Therefore, although the PPS is impaired by altered integrity of the body-brain connections, top-down mechanisms related to action representation may help to restore it. Further studies are necessary to better understand whether the sight of limbs in motion is sufficient or if actual movement is necessary.

## Methods

### Participants

Two groups age and gender-matched people participated in Experiment 1. The PCL group was made up of 18 paraplegic patients (AIS level: A, NLI: < C7, age mean (SD): 43 (10), range: 25–61, 2 females) and 18 neurologically healthy people (age mean (SD): 38 (14), range: 24–64, 6 females) served as controls (C). All participants had normal or corrected-to-normal vision. 14 PCL patients who participated in Experiment 1 also took part in Experiments 2 and 3. Clinical and demographic data of the PCL patients are shown in Table 1. The study was approved by the Ethics committee of the Province of Verona (Prot. N. 40378) and was conducted in accordance with the ethical standards of the 2013 Declaration of Helsinki. All participants gave their written informed consent.

### Apparatus

The hand-made apparatus used in the experiment is shown in Fig. 1. Panel A shows a participant seated in the chair, with the feet positioned on a wooden frame (80 × 38 cm, tilted at 30° from the vertical plane, Panel 1B) where the LEDs were positioned and the two feet-compartments (each 15 × 8 cm, distance 22 cm, shown also in panel C), and holding in the hands the two tactile stimulators (also shown in panel D). Note that the four white LEDs placed in proximity of the four inner corners of the feet compartments (see Fig. 1B), were used as distractor stimuli. One red and one yellow LED placed at the centre of the wooden frame were used as a fixation point and a control light respectively (further details are provided in SM, S4.1).

### Procedure

In Experiments 1 and 3, participants were seated on a plastic chair with the wooden frame on the floor in front of them. In Experiment 2, the frame was placed on a table. In Experiments 1 and 3, the distance of the plastic chair from the wooden box was adjusted according to the length of each participant’s legs (never less than 30 cm). This allowed them to place their feet inside the feet-compartments and maintain a comfortable position. In Experiment 2, the wooden frame was placed at around 15 cm from participants in the “Within Compartments” condition and at about 40 cm from them in the “Outside Compartments” condition. In all the experiments participants held two tactile stimulators (one in each hand) between their index fingers and thumbs, with their index finger on the top and their thumb on the bottom of the device. They were asked to keep their feet and hands (which had been covered with two black cloths) as still as possible. The experimental task was then explained to the participants. In each trial, a different combination of tactile stimulation and visual distractors was administrated. Tactile stimuli (three 50 ms vibrations separated by 50 ms gaps, total duration 250 ms) were applied either on the upper or lower parts of the stimulation device (index or thumb respectively, in the left or right hand, see Fig. 1D). Visual distractor stimuli (3 × 50 ms separated by 50 ms gaps) were displayed close to one of the two feet-compartments, either at the top or the bottom (Fig. 1B,C). As greater CCEs have been shown when visual stimulation starts before the tactile stimulation, the former started 30 ms before the latter^62^. The four positions relating to the tactile stimulations and the visual distractors were randomly associated.

The participants were asked to look at the fixation LED and verbally report where they felt (“high” or “low”) the tactile stimulus irrespectively of the hand, as fast as possible. They were told to ignore the visual distractors.

The verbal response options were selected to avoid fricative consonants in order to facilitate pronunciation and avoid prolonged responses which would make RT computation difficult. There are fricative consonants in the Italian words for *above* (“sopra”) and *below* (“sotto”) so the response options were: i) “TAH” when the tactile sensation was felt on the index finger (high position) and ii) “TOH” when it was felt on the thumb (low position), irrespective of whether the stimulus was on the left or the right.

In order to ensure that participants focused on the centrally positioned LED, they were instructed to answer “LUCI” (the Italian word for “lights”) whenever they saw the yellow LED flashing (control condition), and to make sure that they paid attention to the tactile stimuli, false stimulations were administered. False stimulations where composed by a distractor light flashing without tactile stimulation. In these trials the expected response was: “*Niente*” (the Italian word for “nothing”).

Before the experimental session, about 20 preliminary trials were administered to ascertain that the participant had understood the requirements of the task.

### Sense of Ownership Questionnaire

In order to explore potential changes in the participant’s sense of ownership towards the real and fake feet, a questionnaire was administered in Experiment 1 before and after all the conditions. The statements had been derived from a 27-item questionnaire previously used by Longo and colleagues^39^ which investigated: i) Embodiment of rubber hand; ii) Loss of own hand; iii) Movement and iv) Affective feelings. The items with the highest loadings in the original version (two statements for each component) were selected for our version and adapted (see SM, S4.2), after a process of English-Italian back-translation. Participants indicated their degree of agreement by means of a 10-point Likert scale (1 = do not agree at all; 10 = completely agree). To comply with the requirements of our study, the 4 components were renamed Sense of Embodiment, Loss of own feet, Perceived Movement of lower limbs and Compliance.

### Data handling

A preliminary check of individual performance was made to exclude those participants with scores ≤44% (the chance level without control trials). By means of a script in R^63^ and the *tuneR*^64^ package, the RTs from the audio files (WAV format at 8000 Hz) were computed. For each participant, RTs for incorrect answers were rejected (mean ± SD of rejected trials for participant: Experiment 1 = 7 ± 9.2%; Experiment 2 = 6.33 ± 7.7%; Experiment 3 = 8.08 ± 6.77%).

An assessment of the CCE for Ipsilateral and Contralateral trials and for each condition was subsequently computed for each participant as follows:


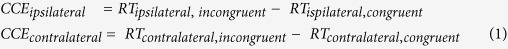


Scores for each questionnaire component in Experiment 1 were obtained by averaging the two item scores.

The Bayesian analysis (computed by means of the R framework for statistical computing) was based on the studies of Bayes (1763)^65^ and Laplace (1825)^66^. This analysis starts from a prior distribution (the mathematical representation of a hypothesis) and computes the likely distribution (using the data on the prior model). The result shows the posterior distribution of the parameters of interest.

In order to perform the Bayesian analysis with R, the *rjags*^67^ package was used to connect it to JAGS^68^ which is a GNU software was used to perform Markov Chain Monte Carlo (MCMC) simulations via the Gibbs sampling algorithm^69^. A total of 10,000 samples were drawn after 1000 burn-in samples for each of 3 chains (for a total of 30,000 samples) for each Bayesian Analysis.

## Additional Information

**How to cite this article**: Scandola, M. *et al.* Spinal cord lesions shrink peripersonal space around the feet, passive mobilization of paraplegic limbs restores it. *Sci. Rep.* **6**, 24126; doi: 10.1038/srep24126 (2016).

## Supplementary Material

Supplementary Information

## Figures and Tables

**Table 1:** Clinical and demographic data for the PCL group.

ID	Age	Gender	NLI	AIS	Experiments
1	60	M	T6	A	S1, S2, S3
2	35	M	T4	A	S1, S2, S3
3	25	F	T6	A	S1, S2, S3
4	53	F	T10	A	S1
5	34	M	T10	A	S1, S2, S3
6	44	M	T8	A	S1
7	33	M	T4	A	S1, S2, S3
8	60	M	T4	A	S1, S2, S3
9	47	M	T11	A	S1, S2, S3
10	54	M	L3	A	S1, S2, S3
11	39	M	T7	A	S1, S2, S3
12	39	M	T1	A	S1, S2, S3
13	39	M	T6	A	S1
14	61	M	T4	A	S1
15	30	M	T4	A	S1, S2, S3
16	47	M	T6	A	S1, S2, S3
17	38	M	T1	A	S1, S2, S3
18	39	M	T4	A	S1, S2, S3

All participants were right handed. NLI = Neurological Level of Injury, AIS = ASIA Impairment Scale (A = complete lesion), Experiments = Experiments in which the subject participated.

**Table 2:** Mean and Standard Deviation values for the Sense of Ownership Questionnaire in Experiment 1.

			Emb.	Loss	Perc.	Com.
Control	Void	Before	1 ± 0	1.44 ± 0.86	1.22 ± 0.43	7.56 ± 1.82
		After	1 ± 0	1.44 ± 0.86	1 ± 0	7.78 ± 1.66
	Fake	Before	1 ± 0	1.67 ± 1.28	1 ± 0	8.11 ± 1.57
		After	1.89 ± 1.71	1.67 ± 1.28	1.67 ± 1.28	8 ± 1.61
	Real	Before	9.56 ± 0.86	1.11 ± 0.32	1.67 ± 1.28	8.11 ± 1.49
		After	8.89 ± 2.14	1.78 ± 1.26	1.44 ± 0.86	7.56 ± 1.82
Paraplegic	Void	Before	1 ± 0	2.5 ± 2.68	1 ± 0	7 ± 2.47
		After	1 ± 0	2.22 ± 2.53	1 ± 0	7.67 ± 1.85
	Fake	Before	1 ± 0	2.61 ± 2.55	1 ± 0	7.44 ± 2.18
		After	1 ± 0	2.39 ± 2.62	1.06 ± 0.24	7.44 ± 2.09
	Real	Before	7.72 ± 3.06	1.44 ± 1.04	1.72 ± 2.16	7.67 ± 2.06
		After	8.17 ± 3	1.33 ± 0.77	1.78 ± 2.37	7.78 ± 2.07

Values are reported for each Component (Emb. = Embodiment of own feet, Loss = Loss of own feet, Perc. = Perceived Movements and Com. = Compliance), Group (Control and Paraplegic), Condition (Void, Fake and Real) and Times (Before the execution of the trials or After).
