# Supplementary material for: Spinal cord lesions shrink peripersonal space around the feet, passive mobilization of paraplegic limbs restores it
Source: Sci Rep. 2016 Apr 6;6:24126. doi: 10.1038/srep24126 (PMC4822176; doi:10.1038/srep24126)
Supplement: Supplementary Information [file srep24126-s1.pdf]

Spinal cord lesions shrink peripersonal space around the feet, passive mobilization of paraplegic limbs restores it

Authors:

Michele Scandola[1,2]*, Salvatore Maria Aglioti[3,2], Claudio Bonente[1], Renato Avesani[4], Valentina Moro[1]*

[1]NPSY-Lab.VR, Department of Philosophy, Education and Psychology. University of Verona, Verona I-37129, Italy

[2]IRCCS, Fondazione Santa Lucia, Rome I-00179, Italy

[3]Department of Psychology, University of Rome “Sapienza”, Rome I-00185, Italy

[4]Department of Rehabilitation, Sacro Cuore - Don Calabria Hospital, Negrar I-37024,Verona,Italy

*michele.scandola@univr.it; *valentina.moro@univr.it

Supplemental Figures

S1 The Space product method for CCE data

By means of a transdimensional hierarchical Bayesian linear regression, the Space Product Method (SPM) was applied directly to CCE indexes as follows:

(1) $$CCE_{index} = CCE_{ipsilateral} - CCE_{contralateral}$$

Using this index, if a PPS representation is present it should be greater than 0 and if a PPS representation is not present it should be equal to 0. In order to set credible values, we selected the means and standard deviations using the actual data of the experiments.

In these models, the intercept and an estimation of the condition for each experimental cell were used as Fixed factors, while an estimation of the contribution of the participant was included as a Random factor.

Two categorical distributions were used as hyperpriors (prior distributions of prior distributions). One distribution was used to select the parameter values that corresponded to the various different hypotheses for the Control group. The other categorical distribution was used to select the hypothesis for the Paraplegic Group.

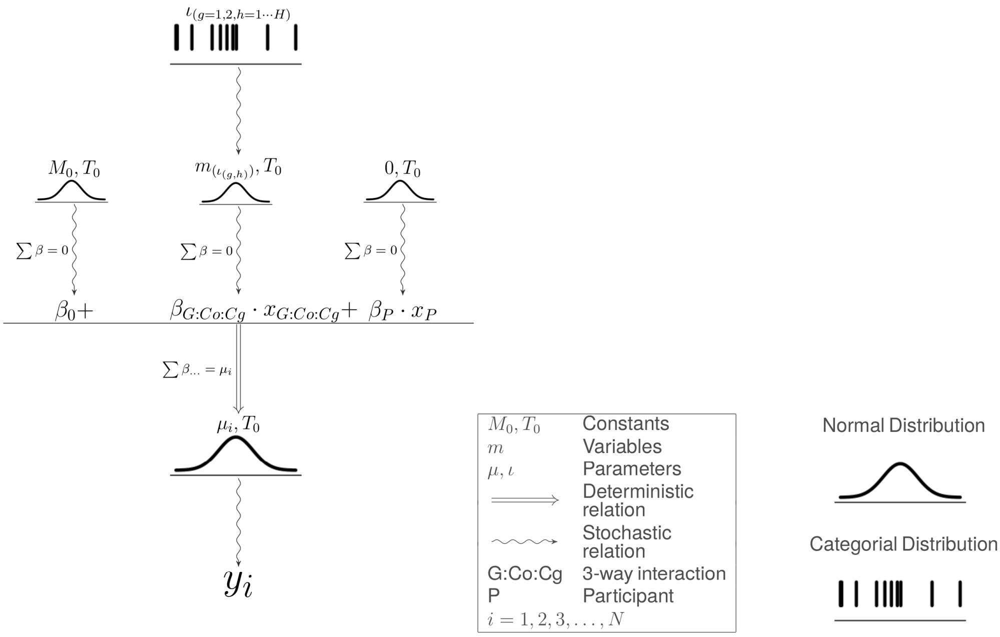

Figure S1 *The Hierarchical Bayesian Model graphic prior to representation for model comparison. The prior parameters for each categorical distribution were set at the same probability. Only the mean of the 3-way interaction component could change according to the categorical distribution estimation. Starting from the top, the Categorical distribution will give stochastic distributions providing estimates for two different ι for Experiment 1: one for the Paraplegics and one for the Control group. For Experiment 2 there was a single ι index and for Experiment 3 two ι indexes, one for the Motion Group and one for the No-motion group. Each value of ι is an index to a different hypothesis among the 8 (Experiment 1) or 4 (Experiments 2 and 3) hypotheses. The hypothesis will give a different value to the variable m, i.e. the mean of the Normal distribution characterizing the β of the condition, reported in the main text of the article. The sum of the βs gives us the mean of the Normal distribution, providing an estimate of the CCE (yi).*

S2 The Space product method for Questionnaire analysis

A Bayesian model selection was applied by means of the Space Product Method. To account for the ordered and categorical nature of the data, a Bayesian hierarchical logit regression model was used (Agresti, 2010, pp 327). The independent variables considered were: Group (Control, Paraplegics), Condition (Real, Fake and Void) and Time (Before or After the experimental session). The dependent variables were the subjective answers of the participants. As in the SPM method for the CCE data used in the previous model (S1), even in this model the intercept and an estimation of the condition for each experimental cell were used as Fixed factors, while an estimation of the contribution of the participant was included as a Random factor.

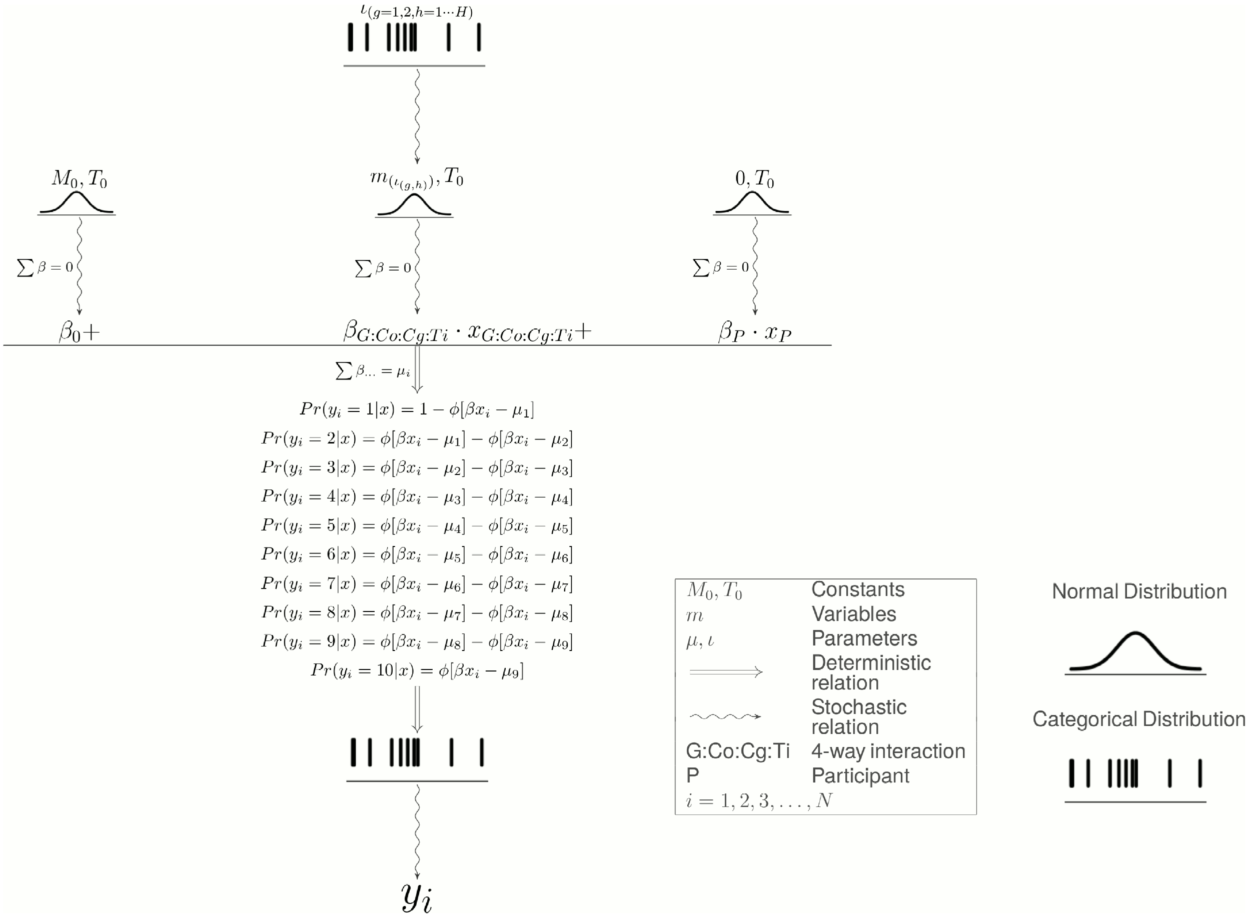

Figure S2. The graphic representation of the hierarchical Logit Bayesian Model prior for model comparison. The prior parameters for each categorical distribution were set at the same probability. Only the mean of the 4-way interaction component could change, according to the estimate of the Categorical distribution. Each value of ι is an index to one hypothesis among the 22 hypotheses. Each hypothesis gives

a different value to the variable m, i.e. the mean of the Normal distribution characterizing the β of the condition. Within the model a β for the Intercept and for the Subject are also estimated. The sum of all the β estimates will give the mean of a Normal cumulative distribution (ϑ). From this distribution the probabilities of each point on the Likert scale are computed. These probabilities are used in the Categorical distribution that is a stochastic approximation of the dependent variable y, namely the score of our questionnaire.

S3 Frequentist analyses

For the CCE analyses, a mixed-effect ANOVA was applied to the data by means of the *afex* package [1] (between-subject factor: group, within-subject factors: condition and left/right congruency). Pairwise t tests with Bonferroni correction were then used to investigate the data. Bootstrap versions of the tests were further applied.

For the questionnaire data in Experiment 1, ordered logistic regressions [2] were used. These are particularly suited to the analysis of ordinal multinomial data as they take into account their ordinal nature but also allow for a complex analysis such as an ANOVA. The analysis was computed by means of the *rms* package [3] to fit the Four models, with one model for each component of the questionnaire (Sense of Embodiment, Loss of own feet, Perceived Movement of lower limbs and Compliance). Each model took into account the Group factor (Control, Paraplegics), the Condition factor (Real, Fake and Void) and the Time factor (Before or After the experimental session).

S3.1 Frequentist Analysis of CCE data for Experiment 1

Effect	Df	MSE	F	η^2	*P*	*p* boot	CI boot
Group	1, 34	9571.15	0.24	.007	.62	.62	0.00 – 3.67
Condition	1.82, 61.82	1671.61	1.26	.04	.29	.20	0.24 – 12.00
**Congruency**	**1, 34**	**2729.74**	**42.73**	**.56**	**<.0001**	**.001**	**19.70 – 74.78**
Group:Condition	1.82, 61.82	1671.61	0.70	.02	.49	.55	0.11 – 8.38
Group:Congruency	1, 34	2729.74	0.06	.002	.81	.80	0.00 – 3.50
Condition:Congruency	1.86, 63.19	890.02	1.72	.05	.19	.19	0.26 – 12.62
**Group:Condition:Congruency**	**1.86, 63.19**	**890.02**	**4.01**	**.11**	**.03**	**.03**	**1.27 – 21.55**

Table S3.1. Results from the ANOVA on CCE data from Experiment 1. For bootstrapped values, there were 1000 repetitions.

A post-hoc analysis on the CCE via pairwise t-tests using a Bonferroni correction showed that there was a statistically significant difference (p = 0.0043) when the CCEs of the Control group were compared in the Real condition Ipsilateral vs. Contralateral.

S3.2 Frequentist Analysis of Questionnaire data for Experiment 1

Effect	Df	χ^2	*P*
Group	6	4.04	0.6718
**Condition**	**8**	**28.98**	**0.0003**
Time	6	0.47	0.9982
Group:Condition	4	0.44	1.0000
Group:Time	3	0.01	0.9309
Condition:Time	4	0.01	1.0000
Group:Condition:Time	2	0.00	0.9982

Table S3.2.1. Ordered logistic Regression results relating to the Questionnaire Data for the "Sense of Embodiment" component.

Bonferroni pairwise Wilcoxon tests carried out on the Condition effect show that in the Real condition answers were significantly higher than in the other conditions (both ps < .0001).

Effect	Df	χ^2	*P*
Group	6	5.03	0.5397
Condition	8	6.44	0.5981
Time	6	3.05	0.8024
Group:Condition	4	2.15	0.7079
Group:Time	3	1.61	0.6571
Condition:Time	4	1.91	0.7519
Group:Condition:Time	2	0.58	0.7483

Table S3.2.2. Ordered logistic Regression results relating to the Questionnaire Data for the "Loss of own feet" component.

No significant changes in the "Loss of own feet" component.

Effect	Df	χ^2	*P*
Group	6	3.23	0.7796
Condition	8	0.75	0.9994
Time	6	0.10	1.0000
Group:Condition	4	0.52	0.9714
Group:Time	3	0.02	0.9991
Condition:Time	4	0.06	0.9996
Group:Condition:Time	2	0.01	0.9966

Table S3.2.3. Ordered logistic Regression results relating to the Questionnaire Data for the "Perceived movements" component.

No significant changes in the "*Perceived movements*" component.

Effect	Df	χ^2	*P*
Group	6	2.29	0.8914
Condition	8	2.82	0.9449
Time	6	1.76	0.9404
Group:Condition	4	0.72	0.9209
Group:Time	3	1.30	0.8694
Condition:Time	4	0.93	0.8614
Group:Condition:Time	2	0.26	0.8798

Table S3.2.4. Ordered logistic Regression results relating to the Questionnaire Data for the "Compliance" component.

No significant changes in the "*Compliance*" component.

S3.3 Frequentist Analisys of CCE data for Experiment 2

Effect	Df	MSE	F	η^2	*P*	*p* boot	CI boot
Condition	1, 13	9041.23	1.40	.10	.26	0.8342	0.00 – 8.13
Congruency	1, 13	4301.85	4.13	.24	.06	0.2547	0.06 – 14.92
**Condition:Congruency**	**1, 13**	**973.28**	**36.42**	**.74**	**< .0001**	**0.001**	**14.80 – 83.69**

Table S3.3. Results from the ANOVA on the CCE data relating to Experiment 2. For bootstrapped values there were 1000 repetitions.

Bonferroni corrected post-hoc t-tests showed that the only significant difference between Ipsilateral and Contralateral trials was in the PPS condition (p = .0049).

S3.4 Frequentist Analysis of CCE data for Experiment 3

Effect	Df	MSE	F	η^2	*P*	*p* boot	CI boot
Group	1, 12	9864.89	3.56	.23	.08	0.0779	0.04 – 13.32
Congruency	1, 12	2460.97	1.51	.11	.24	0.2457	0.00 – 8.62
**Group:Congruency**	**1, 12**	**2460.97**	**5.39**	**.31**	**.04**	**0.0370**	**0.25 – 19.43**

Table S3.4. Results from the ANOVA on the CCE data relating to Experiment 3. For bootstrapped values there were 1000 repetitions.

Bonferroni corrected post-hoc t-tests showed that the only significant difference between Ipsilateral and Contralateral trials was in the Motion group ($p = .048$, one-tail).

S4. Supplemental Experimental Procedures

S4.1. Apparatus

The apparatus was designed by MS. The instrumentation for the cross-modal paradigm was composed of the following:

• Two vibrating tactile stimulators for the hands that vibrate on the thumb and index finger

• a wooden frame for the feet and the visual stimuli (six LEDs)

• a microcontroller to control the tactile stimulators and the visual distractors

• a notebook to control the microcontroller and to record the vocal data collected from the microphone

• a plastic chair

• two fake feet

Each tactile stimulator consisted of two Dayton Audio DAEX25 Sound Exciter Pairs. The tactile stimulators were small cylinders that can independently vibrate on either side, at the contact points where the participant held the stimulator. The tactile stimulators were designed to be held by the index finger and thumb.

A wooden frame was prepared with two feet-compartments (each 15 × 8 cm). The inner borders of the compartments were 22 cm apart. Four white LEDs to be used as distractor stimuli were mounted near the inner corners of the feet-compartments. (Figure 1A, 1B, 1C). One red and one yellow LED were positioned at the centre of the wooden frame in order to provide a fixation point and a control light, respectively. The apparatus was covered by a black rubber cloth to ensure that the LED lights contrasted with the background. The wooden frame was designed to be placed on the floor and tilted so that the feet-compartments were at an angle of 50 degrees. This design allowed the feet-compartments and the LED lights to be clearly visible.

Both the LED lights on the wooden frame and the tactile stimulators were connected to a microcontroller (Arduino Uno TM , www.arduino.cc). This was programmed using the "Processing" programming language (www.processing.org) which controlled the individual experimental trials.

The microcontroller was connected to a Notebook computer (ASUS X53S) that recorded the data in Microsoft Visual C# ™ (www.microsoft.com), correctly randomized the individual trials and recorded the vocal responses via the microphone. The tactile stimulators produced a small auditory tone which was masked by white noise on an mp3 player (played into headphones) in order to avoid a confounding effect.

The tactile stimulators were attached to flexible metal bands and connected to a comfortable plastic chair.

S4.2. Sense of Ownership Questionnaire

Sense of Embodiment

1. *I had the sensation that what is inside the feet-compartments belongs to me.* (In italian: '*Ho la sensazione che ciò che c'è nella pedana mi appartenga*').
2. *It seemed like my real feet were inside the feet-compartments. (In Italian* 'Mi sembra che ciò che c'è nella pedana siano i miei piedi').

Loss of own feet

1. *It seemed like I couldn't really tell where my feet were. (in Italian:* 'Mi sembra di non saper dire dove sono i miei piedi').
2. *It seemed like my feet had vanished. (In Italian:* 'Mi sembra come se i miei piedi fossero spariti').

Perceived Movements

1. *It seemed like my own feet were moving. (In Italian:* 'Mi sembra come se i miei piedi si stessero spostando').
2. *It seemed like what is inside the feet-compartments was moving towards me. (In Italian:* 'Mi sembra come se ciò che c'è nella pedana stesse spostandosi verso di me').

Compliance

1. *I found the experience enjoyable.*(*In Italian:* Mi sto divertendo).
2. *I found the experience interesting.* (L'esperienza mi sta interessando).

References

1. Singmann, H. afex: Analysis of Factorial Experiments. (2014).

2. Agresti, A. *Categorical Data Analysis*. (John Wiley & Sons, Inc., 2002).

3. Harrell, F. E. J. rms: Regression Modeling Strategies. (2014).
